# Systematic Identification of Protein Targets of Sub5 Using *Saccharomyces cerevisiae* Proteome Microarrays

**DOI:** 10.3390/ijms22020760

**Published:** 2021-01-13

**Authors:** Pramod Shah, Chien-Sheng Chen

**Affiliations:** 1 Graduate Institute of Systems Biology and Bioinformatics, and Department of Biomedical Sciences and Engineering, College of Health Sciences and Technology, National Central University, Jhongli 32001, Taiwan; prokonp@gmail.com; 2 Institute of Molecular Biology, Academia Sinica, Taipei 115, Taiwan; 3 Department of Food Safety/Hygiene and Risk Management, College of Medicine, National Cheng Kung University, Tainan 701, Taiwan

**Keywords:** Sub5, antifungal activity, antimicrobial peptides (AMPs), proteome microarray, target identification

## Abstract

Antimicrobial peptides (AMPs) are intensively studied in terms of alternative drugs. Sub5 is a synthetic 12-mer AMP with substitutions of five amino acids of bactenecin 2A (Bac2A), a linear-ized bactenecin variant of bovine. Sub5 is highly effective against fungi with an ability to trans-locate cell membrane, but its targets are unknown. Systematic analysis of Sub5 targets will facil-itate our understanding on its mechanism of action. In this study, we used high-throughput *Saccharomyces cerevisiae* proteome microarrays to explore the potential protein targets of Sub5. The screening results showed 128 potential protein targets of Sub5. Bioinformatics analysis of protein targets of Sub5 revealed significant gene ontology (GO) enrichment in actin related pro-cess of “actin filament-based process”, “actin filament organization”, “actin cortical patch or-ganization”, regulation of “actin filament bundle assembly”. Moreover, the other enriched cat-egories in GO enrichment mostly contained actin associate proteins. In total, 11 actin-associated proteins were identified in the protein targets of Sub5. Protein family (PFAM) enrichment anal-ysis shows protein domain enriched in actin binding, i.e., “Cytoskeletal-regulatory complex EF hand (helix E-loop-helix F motif)”. Being consistent with GO analysis, Search Tool for the Re-trieval of Interacting Genes/Proteins (STRING) analysis of the protein targets of Sub5 showed ac-tin network with involvement of 15 protein targets. Along with actin-network, STRING analysis showed protein–protein interaction network in ribonucleoprotein, transcription and translation, chromosome, histone, and ubiquitin related, DNA repair, and chaperone. Multiple Expression motifs for Motif Elicitation (MEME) suite provided a consensus binding motif of [ED][ED]EEE[ED][ED][ED][ED][ED], in total of 75 protein targets of Sub5. This motif was present in 9 out of 15 actin-related proteins identified among protein targets of Sub5.

## 1. Introduction

Antimicrobial peptides (AMPs), a hope for the next antimicrobial agents, have activities against bacteria (Gram-negative and Gram-positive), fungus, enveloped virus, as well as cancer cells. AMPs are small peptide molecules, which are omnipresent in organism tissues, where they play a key role in non-specific defense systems [1]. Mucosal epithelial cells (Paneth cells) and phagocytes (macrophages, neutrophils, and NK cells) are a rich source of AMPs [2]. Upon infection, AMPs are synthesized much earlier than immunoglobulins from adaptive immune response [3]. Usually, AMPs are 12 to 46 amino acids long with diverse sequences, structures, and function. The cationic AMPs mostly contain a net positive charge of +2 or more at neutral pH (in presence of arginine or lysine amino acid residues in their sequence) [4] and amphipathic structures with hydrophilic and hydrophobic domain. Cationic AMPs facilitates electrostatic interaction with anionic phospholipids membrane whereas their amphipathic structures incorporate into hydrophobic backbone of cytoplasmic membranes that lead to the formation of pores and channels. These pores and channels cause leakage of cell contents and eventually lead to cell death [5]. AMPs also exert non-membrane lysis mechanism of actions where they target several intracellular molecules [1].

Mostly, the known targets of conventional antibiotics are cell wall and intracellular macromolecules. Cell envelop is the major barrier that abate function of antibiotics, moreover, by modifying cell envelop, microorganisms are able to develop drug resistance. AMPs, on the other hand, have a wide range of targets that not only lyse membrane, but also easily translocate these membranes and targets on intercellular targets. These multi-targeting mechanisms of AMPs are a potential for the development of novel therapeutics agents.

Sub5 (RRWKIVVIRWRR), a synthetic variant of Bac2A with substitutions of five amino acids of a total of 12 amino acid sequence, has a net charge of +6 and 50% of hydrophobicity [6]. Bac2A (RLARIVVIRVAR) is a linearized variant of loop-shaped bactenecin (RLCRIVVIRVCR), a 12-mer proline/arginine rich AMPs obtained from bovine neutrophils, with increased Gram-positive activities [7]. The positive net charge of Bac2A and bactenecin is reported to +4 and +3, respectively [7]. The loop shaped bactenecin targeted on cell membrane, while the liner form had different mechanisms of action. Further, the substitution of five amino acids of Bac2A to produce Sub5 not only improved the antimicrobial activities, but also lowered the minimal inhibitory concentration (MIC) against bacteria as well as fungi. Same MIC of 4 µg/mL is reported for Sub5 against *Escherichia coli* and *Candida albicans* [6]. Another study also replicated these findings of the same MIC of Sub5 against bacteria and fungi [6,8]. Moreover, Sub5 was reported to target cytoplasmic membrane as well as enter the cytoplasm with prolong exposure, and suggested to have intracellular targets [8]. This make Sub5 a potential and promising antifungal agent as an alternative therapeutic. Several AMPs, including Sub5, are identified as potential antifungal agents; however, their targets are unknown. In the lack of target, the modes of action of Sub5 is poorly understood. To explore the entire protein targets of Sub5, *Saccharomyces cerevisiae* proteome microarrays were employed. *Saccharomyces cerevisiae* proteome microarrays provided systematical discovery of direct protein targets of Sub5 from Sub5-protein interactions. Proteome microarrays that contain the entire proteomes of particular organism (*Escherichia coli*, *Saccharomyces cerevisiae,* and human) are high-throughput tools, recently used for the identification of, e.g., protein–protein, protein–drug, protein–DNA, and other interactions [9,10]. Previously, we discovered the protein targets of two novel antifungal AMPs (Lfcin B and Histatin-5) using *Saccharomyces cerevisiae* proteome microarrays [11]. Moreover, by using *Escherichia coli* proteome microarrays, in past, we successfully identified the protein targets of four AMPs, which are LfcinB, proline-arginine rich AMP (PR-39), hybrid of pleurocidin and dermaseptin (P-Der), and Bactenecin 7 (Bac 7) [12,13,14]. In this study, 128 protein targets of Sub5 were systematically identified through *Saccharomyces cerevisiae* proteome microarrays. Bioinformatics analysis were performed to identify the enrichment, protein–protein interactions, and motif analysis.

## 2. Results

### 2.1. Saccharomyces Cerevisiae Proteome Microarrays Assays of Sub5

**Figure 1:**
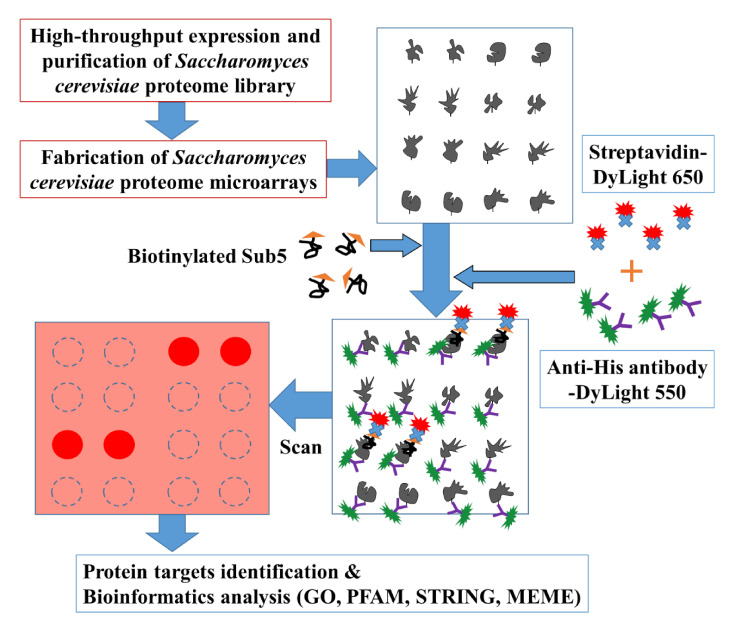
Schematic diagram for identifying the protein targets of Sub5 using *Saccharomyces cerevisiae* proteome microarrays. To find the targets, biotinylated Sub5 was probed on *Saccharomyces cerevisiae* proteome microarrays and later probed with streptavidin-DyLight 650 and anti-His antibody-DyLight 550 for the detection of signal of biotinylated Sub5 that interacted with *Saccharomyces cerevisiae* proteins, and proteins containing His tag to represent the protein amount of each proteins on *Saccharomyces cerevisiae* proteome microarrays, respectively. The protein targets of Sub5 were identified using several cutoff parameters and the final list of protein targets of Sub5 were analyzed with bioinformatics tools gene ontology (GO), Protein family (PFAM), Search Tool for the Retrieval of Interacting Genes/Proteins (STRING), and Multiple Expression motifs for Motif Elicitation (MEME).

To systematically pinpoint the target proteins of Sub5, the high throughput *Saccharomyces cerevisiae* proteome microarrays containing, in total, ~5800 individually purified *Saccharomyces cerevisiae* proteins spotted on the aldehyde coated glass slides, were employed. Figure 1 depicts the overall schematic diagram of current study. Briefly, *Saccharomyces cerevisiae* proteome microarrays were, firstly, probed with biotinylated Sub5 and, secondly, with streptavidin-DyLight 650 and anti-His antibody-DyLight 550. Streptavidin-DyLight 650 was utilized for the detection of biotinylated Sub5 bound to specific *Saccharomyces cerevisiae* proteins on *Saccharomyces cerevisiae* proteome microarrays. Moreover, anti-His antibody-DyLight 550 was used to detect 6× His tag of the individually purified *Saccharomyces cerevisiae* proteins. Here, the signal of anti-His antibody-DyLight 550 represents the total protein amount of the individual *Saccharomyces cerevisiae* protein on the *Saccharomyces cerevisiae* proteome microarray. *Saccharomyces cerevisiae* proteome microarray contains duplicate spot of each *Saccharomyces cerevisiae* proteins and, in total, triplicate *Saccharomyces cerevisiae* proteome microarray assays were conducted for systematic identification of Sub5 protein targets.

### 2.2. Statistical Analysis of Protein Targets of Sub5

**Figure 2:**
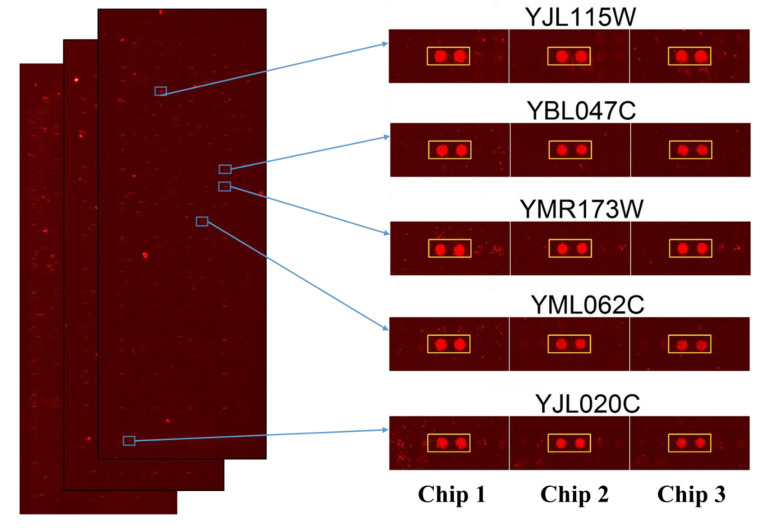
Image of Sub5 targets on *Saccharomyces cerevisiae* proteome microarrays and the enlarge images of representative protein targets. Images of *Saccharomyces cerevisiae* proteome microarrays probed with Sub5 and the representative protein target of Sub5 is shown in entire proteome, as well as individual enlarge images from the triplicate proteome microarray assays. The two sport represent the duplicate protein probed on *Saccharomyces cerevisiae* proteome microarrays (Chips). Chip 1, 2, and 3 represent the triplicate *Saccharomyces cerevisiae* proteome microarrays employed for Sub5 probing to systematically identify the protein targets. Red spots represent signal of Sub5 bound to immobilized Saccharomyces cerevisiae proteome.

After performing the *Saccharomyces cerevisiae* proteome microarrays assays in triplicate, the data were individually exported in GenePix Pro Results (GPR) files and analyzed together in excel with several cutoff parameters. Firstly, median scaling normalization were used for the normalization of triplicate microarrays assays. Then, signal cutoff, coefficient of variable cutoff, ratio cutoff, and finally, the local cutoff parameter of signal greater than mean, plus two standard deviations, were used for the selection of protein targets of Sub5 from *Saccharomyces cerevisiae* proteome microarrays. The parameter and selection processes were detailed in the Section 4. The 131 protein targets of Sub5 were identified from *Saccharomyces cerevisiae* proteome microarrays by using several statistical parameters. The statistically identified protein targets of Sub5 from the triplicate *Saccharomyces cerevisiae* proteome microarrays were individually judged by eye to confirm the identified protein targets were the outstanding hits of Sub5. By removing the protein targets of Sub5 that did not pass the eye estimation test, a potential list of Sub5 targets was finalized that contain 128 protein targets. The entire protein targets of Sub5 identified from *Saccharomyces cerevisiae* proteome microarrays are shown in Supplementary Table S1. The images of triplicate *Saccharomyces cerevisiae* proteome microarrays probed with Sub5, as well as the amplified image of representative protein targets of Sub5 (in duplicate) obtained from three independent *Saccharomyces cerevisiae* proteome microarray assays are depicted in Figure 2. Supplementary Figure S1 shows the enlarged images of all of the 128 protein targets of Sub5 from triplicate *Saccharomyces cerevisiae* proteome microarrays.

### 2.3. Bioinformatics Enrichment Analysis in Gene Ontology of the Protein Targets of Sub5

To identify the enriched categories in the protein targets of Sub5 having similar function, Gene Ontology (GO) enrichment in different categories (biological process, molecular function, and cellular component) were analyzed. The Database for Annotation, Visualization, and Integrated Discovery (DAVID), version 6.8 [15], with functional annotation tools, was used for Gene Ontology (GO) enrichment analysis applying stringent *p*-value cutoff of 0.05.

**Figure 3:**
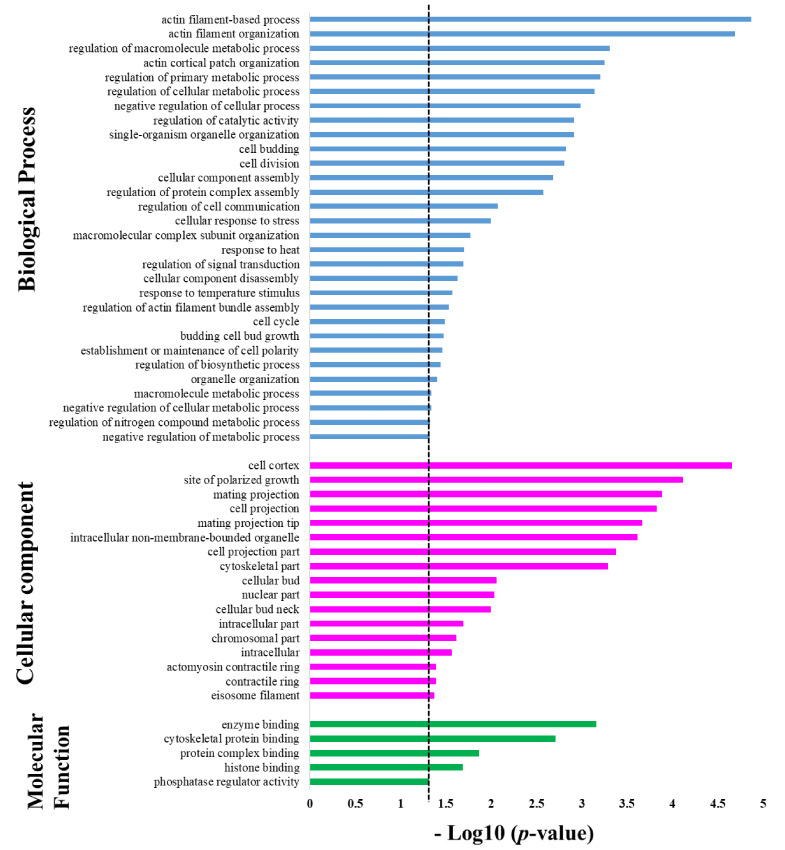
Gene ontology enrichment in biological process, cellular component, and molecular function. The signification enrichment in different categories of gene ontology of protein targets of Sub5 obtained by Saccharomyces cerevisiae proteome microarrays; (*p*-value cutoff of 0.05 was indicated by dotted line). Biological process (blue), cellular component (pink), and molecular function (green).

Protein targets of Sub5 showed significant enrichment in several categories under the GO enrichment class in biological process. The GO biological process enrichment result is depicted in Figure 3 (blue color bar) and details information on number of protein targets involved in specific categories are listed in Table 1. The enrichment results show, in total, four enrichment categories related to actin filaments (i.e., “actin filament-based process”, “actin filament organization”, “actin cortical patch organization”, and regulation of actin filament bundle assembly”). Among 128 protein targets of Sub5, 14 actin-related proteins were identified from the above-mentioned enriched categories for actin filaments. These 14 actin-related proteins are key proteins for several biological processes, thus, it was no surprise to observe the involvement of these actin-related proteins in the other enrichment categories of biological process. For examples, the 14 identified actin-related proteins are involved in several enrichment categories in organization, “single-organism organelle organization”, “cell budding”, “cell division”, “cellular component assembly”, “macromolecular complex subunit organization”, “cellular component disassembly”, “cell cycle”, “budding cell bud growth”, “establishment or maintenance of cell polarity”, “organelle organization”. These actin-related proteins are also involved in several regulatory processes enriched in the GO biological process categories (Figure 3), not only in organization. These includes, “regulation of macromolecule metabolic process”, “regulation of cellular metabolic process”, “regulation of primary metabolic process”, “negative regulation of cellular process”, “regulation of catalytic activity”, “regulation of protein complex assembly”, “cellular response to stress”, “macromolecule metabolic process”, “regulation of biosynthetic process”, and “regulation of nitrogen compound metabolic process”. The result indicates that the protein targets of Sub5 are mostly enriched in actin-related proteins categories of the GO biological process.

Except for actin-related proteins, other cytoskeleton related proteins were also identified among protein targets of Sub5. Two proteins: YGR086C (PIL1) and YPL004C (LSP1), related to cortical structures of eisosome filament proteins that affect plasma membrane organization, were identified. These two proteins were involved in the enrichment categories of “response to heat”, “response to temperature stimulus”, “negative regulation of cellular metabolic process”, “negative regulation of metabolic process” and also in GO biological process enriched categories related to actin filaments (blue color bar in Figure 3).

Two enrichment categories in GO biological processes that did not involve actin-related proteins are “regulation of cell communication” and “regulation of signal transduction”. This indicate that Sub5 also have mechanisms other than actin related functions.

GO class of cellular components showed significant enrichment in several categories, as shown in Figure 3 (pink color bar) and further detailed in Table 2, showing the number of protein involved in each category. Actin-related proteins are highly enriched in biological process, thus, involvement of actin-related proteins in each GO cellular component enrichment category was analyzed. The results were consistent with the biological process analysis, as most of the cellular component enriched categories contain actin-related proteins. The enriched categories are “cell cortex”, “site of polarized growth”, “mating projection”, “cell projection”, “mating projection”, “intracellular non-membrane-bounded organelle”, “cell projection part”, “cytoskeletal part”, “cellular bud”, “cellular bud neck”, “intracellular part”, “intracellular”, “contractile ring”, “actomyosin contractile ring”. Except actin-related, GO cellular component showed enrichment in “eisosome filament”, which is, again, in accordance with the results of biological process enrichment. Moreover, GO cellular component enrichment categories (pink color bar in Figure 3) showed enrichment in two categories not related to actin, these are “nuclear part” and “chromosomal part”, pointing toward the nucleus; chromosomal related proteins are also significant targets of Sub5.

In the GO class of molecular functions, protein targets of Sub5 showed significant enrichment in five categories with *p*-value cutoff of 0.05 (green color bar in Figure 3 and detailed information in Table 3). In “enzyme binding” and “histone binding” categories, actin associated proteins were not identified. Whereas, actin-related proteins of Sub5 protein targets were mostly observed in enrichment categories of “cytoskeletal protein binding” and “protein complex binding”, which were consistent with the biological process and cellular component. Out of four proteins in the “phosphatase regulator activity” enrichment categories, one protein, YAR014C (BUD14), belongs to actin-related proteins, indicating its relation involved to actin-related functions.

### 2.4. Protein Domain Enrichment among the Protein Targets of Sub5

To identify the enrichment in the protein domain, the protein family (PFAM) analysis tool in DAVID was used. The list of protein domain enrichment with *p*-value cutoff of 0.05, along with the numbers and names of proteins involved in each category, is depicted in Table 4. The enriched categories “cytoskeletal-regulatory complex EF hand”, which indicate binding domain or motif with helix-loop-helix structure that control actin cytoskeletal dynamics, “SRC homology 3 domain (SH3 domain)” and “tropomyosin like” domain enrichment is associated with actin-related proteins. As active-related proteins are highly enriched within protein targets of Sub-5, this domain enrichment result is consistent with the GO enrichment finding. The enrichment observed in “eisosome component PIL1” is also consistent with eisosome protein associated categories observed in GO enrichment. Furthermore, the enrichment category, other than actin-related proteins, is represented by significant enrichment in “ubiquitin-conjugating enzyme”, which is in accordance with GO enrichment category results.

### 2.5. STRING Analysis for Interaction between of Protein Targets of Sub5

**Figure 4:**
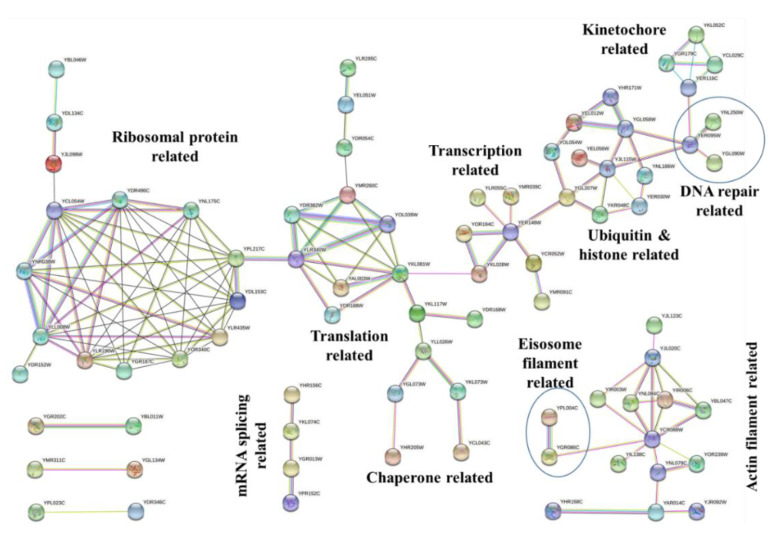
STRING analysis for protein–protein interactions among protein targets of Sub5. Protein targets of Sub5 showed few interaction hubs in STRING analysis. These hubs have functions in ribosome, translation, transcription, chaperone, actin, histone, DNA repair, and kinetochore. This figure was generated by using the STRING database, with high confidence of 0.7 as a cutoff for minimum required interaction score.

To analyze the protein–protein interaction and obtain the significant network hub of proteins among the protein targets of Sub5, STRING database was used [16]. Figure 4 shows the interaction networks among the protein targets of Sub5 by applying high confidence 0.700 cutoff. Based on the GO enrichment results, it was obvious to observe actin related interaction in STRING analysis. STRING also showed an interaction network related to ribonucleoprotein (ribosome-protein), translation, transcription, chaperone, ubiquitin, and histone, DNA repair, and kinetochore. These protein–protein network hubs are in accordance with the GO enrichment results and PFAM enrichment, depicting the protein targets of Sub5 mostly related to actin-related functions, and also few novel functions or complex.

### 2.6. Consensus Motif Search among the Protein Targets of Sub5

**Figure 5:**
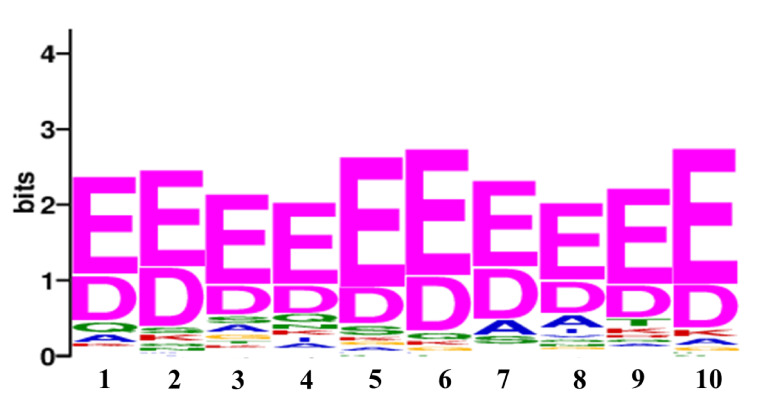
The consensus motif identified in protein targets of Sub-5. Using MEME, the consensus motif [ED][ED]EEE[ED][ED][ED][ED][ED] was identified in 75 protein targets of Sub5 with a value of 8.7 × 10^−25^.

To identify the consensus motif in the protein targets of Sub5, Multiple Expression motifs for Motif Elicitation (MEME) suite was used [17]. The MEME suite analysis showed a consensus motif [ED][ED]EEE[ED][ED][ED][ED][ED], with a motif sequence length of 10 amino acids (Figure 5). This consensus motif was observed in 75 protein targets of Sub5 out of 128 (i.e., ~60% coverage) with a value of 8.7 × 10^–25^. Name of the proteins that have consensus motif are listed in Supplementary Table S2. It is not surprising to notice 9 out of 14 actin-related proteins and two eisosome filament related proteins in the list of 75 proteins of MEME consensus motif. These findings are consistent with the significant enrichment in actin-related functions. Together, this identified consensus motif represents the binding motif of Sub5 to its protein targets.

## 3. Discussion

Increase in immunocompromised patients with several life-threatened diseases, such as cancer, viral infection (HIV), and organ transplantation has significantly increased the incidence of fungal infection [18]. Limited numbers of antifungal drugs with limited spectrum of activities, as well as toxicities to human cells due to an evolutionarily close relationship, and further development of resistance to the existing antifungal drugs, have greatly compromised the heath of humans [19,20]. Thus, alternative antifungal agents are urgently in need.

Consideration of AMPs as antifungal therapeutic agents require high antifungal activity and low hemolytic activity. Sub5 was demonstrated to have no hemolytic activity against horse erythrocytes at a concentration of 256 µg/mL, which is much higher than its MIC [21]. Thus, Sub5 is a safe AMP that can be used as a therapeutic agent. Sub5 was reported with improved antibacterial and antifungal activities [6]. The antibacterial mechanism of action of Sub5 reported in *E. coli* showed intracellularly targeting to ATP and few ATP-dependent proteins, such as DnaK and DNA polymerase [22]. The antifungal mechanism of action of Sub5 is also reported to target intracellular molecules [8], but its target proteins have not been identified. To provide the answer of the potential target proteins of Sub5, in this study, we used *Saccharomyces cerevisiae* proteome microarray that provided systematical screening of Sub5 interaction proteins from the entire *Saccharomyces cerevisiae* proteome in a single assay. *Saccharomyces cerevisiae* proteome microarrays identified, in total, 128 protein targets of Sub5. As ATP and ATP-dependent proteins were reported to be the target of Sub5 (in bacteria), the 128 *Saccharomyces cerevisiae* protein targets of Sub5 were analyzed to find the proteins involved in ATP-dependent functions. In total, 25 protein targets of Sub5 were identified that were involved in ATP binding and ATP-dependent proteins. These ATP binding and ATP-dependent proteins are denoted by the star mark (*) in the list of 128 protein targets of Sub5 mentioned in Supplementary Table S1.

Bioinformatics analysis for enrichment and protein–protein interaction network of protein targets of Sub5 in GO categories and STRING analysis showed significant enrichment in actin-related functions. Actin is a cytoskeleton filament involved in numerous cellular functions that include cell motility, organelle movement, cell signaling, cell division, as well as maintaining cell junctions and cell shape. Targeting actin results in cytoskeleton disorganization that eventually leads to endocytosis, nuclear segregation, hyphal formation causing programmed cell death [23,24]. Actin is a novel target and few actin-binding drugs (e.g., occidiofungin) are being tested to battle in fungal infections [25]. Sub5 protein targets enriched in actin-related processes point out its novel mechanisms in targeting actin organization and regulation, which might result in disorganization of actin filaments, indeed, affecting all of the actin relation processes in cell, and eventually leading to cell death.

**Figure 6:**
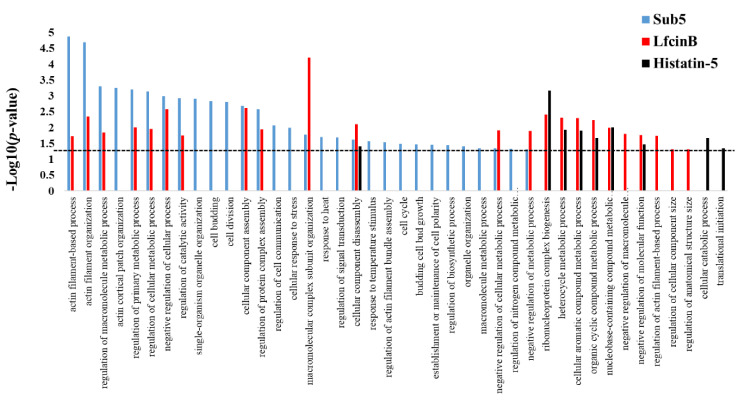
Comparison of the enriched categories in GO biological process for the protein targets of Sub5 (in this study) with Lfcin B and Histatin-5 (in previous study) identified by *Saccharomyces cerevisiae* proteome microarrays. AMPs have their unique sequence, structure, and functions. The enrichment result in the GO biological process of protein targets of Sub5 were compared with previously identified enrichment results in the GO biological process of protein targets of LfcinB and Histatin-5 to observe similarity and difference in the target patterns of AMPs. The *p*-value cutoff of 0.05 was indicated by dotted line.

Previously, we reported the protein targets of antifungal AMPs, such as Lfcin B and Histatin-5 using *Saccharomyces cerevisiae* proteome microarrays [11]. Sub5, Lfcin B, and Histatin-5 are all potential AMPs acting against fungal pathogen; thus, we are interested in knowing the common mechanism in all three AMPs, as well as unique mechanisms that differentiate them from each other. Enrichment analysis in GO biological process for the protein targets of Sub5, Lfcin B, and Histatin-5 were individually perform using DAVID online tools. The obtained GO enrichment results of Sub5, Lfcin B, and Histatin-5 were compared to analyze the unique and common enrichment target patterns of these AMPs. The results as depicted in Figure 6 shows single enrichment category i.e., “cellular component disassembly” to common to all three AMPs (Sub-5, Lfcin B, and Histatin-5). These findings demonstrate that Sub5, Lfcin B, and Histatin-5 have mostly unique target pattern, which might be due to the sequence and structural difference. The total number of unique categories enriched in protein targets of Sub5, Lfcin B, and Histatin-5 are 17, 4, and 2, respectively. These comparison results also showed 12 commonly enriched categories between Sub5 and Lfcin B, such as “actin filament-based process”, “actin filament organization”, “regulation of macromolecule metabolic process”, “regulation of primary metabolic process”, “regulation of cellular metabolic process”, “negative regulation of cellular process”, “regulation of catalytic activity”, “cellular component assembly”, “regulation of protein complex assembly”, “macromolecular complex subunit organization”, and “negative regulation of cellular metabolic process”. Sub5 and Lfcin B showed several common enrichments in actin-related processes, which is in accordance with the total actin-related proteins of 15 and 8 in the entire protein targets of Sub5 and Lfcin B, respectively. The presence of more actin associated proteins make Sub5 a potential target of actin, which can also be noticed by several unique enrichment categories for Sub5 than Lfcin B. This result indicates that Sub5 significantly targets to actin, than Lfcin B. Furthermore, six common enrichment categories between Lfcin B and Histatin-5 are observed here and in our previous study [11].

## 4. Materials and Methods

### 4.1. Expression and Purification of Entire Saccharomyces Cerevisiae Proteome

The entire proteome of *Saccharomyces cerevisiae* containing ~5800 proteins were individually expressed and purified following previous reported protocol [26,27]. *Saccharomyces cerevisiae* proteome library has been constructed with galactose GAL1 promoter, moveable open reading frame, and triple affinity tag (6× His-HA-Glutathione-S-transferase) at C-terminal in a high-copy number URA3 expression vector pBG1805 [28]. For protein expression, these *Saccharomyces cerevisiae* clones stored at −80 °C were grown on SC-URA3-glucose agar plates by incubating at 30 °C for 48 h. Colonies on agar plates were transferred to 96-deep well plates containing SC-URA3-glucose medium, incubated at 30 °C for 24 h. Yeast cells were grown in 12 channel reservoirs containing SC-URA3-raffinose medium, incubated at 30 °C for ~16 h (optical density of 600 nm = 0.6–1.0). Materials were mostly purchased from Sigma Aldrich (Sigma Aldrich St. Louis, MO, USA). To express *Saccharomyces cerevisiae* proteins, a final concentration of 2% galactose was added, and cells were further grown at 30 °C for 4 h. *Saccharomyces cerevisiae* cell pellets were collected by centrifugation of the culture at 4000 rpm for 2 min. Re-suspending the pellet in 800 µL deionized water, the *Saccharomyces cerevisiae* cells were transferred from a 12-channel reservoir to 96-deep well plates. Finally, *Saccharomyces cerevisiae* cell pellets were collected in 96-deep well plates by centrifugation at 4000 rpm for 2 min.

Following standard protocol, the collected *Saccharomyces cerevisiae* cell pellets were used for the protein purification. For cell rupture, 200 µL zirconia beads with 0.7 mm diameter (BioSpec Products Inc. Bartlesville, OK, USA), were loaded in 96-deep well plates containing cell pellets. The 800 µL freshly prepared lysis buffer containing protease inhibitors (50 µM calpain Inhibitor I (LLnL), 1 mM PMSF, 1 µM MG132, and 50× dilution of Roche protease inhibitor) was added to each well, and the 96-deep well plates were incubated on microplate vortex at 4 °C for 30 min. The 96-deep well plates were centrifuged at 4000 rpm for 15 min and supernatant in each well was carefully transferred to new 96-deep well plates. The 100 µL pre-washed glutathione (GSH) Sepharose 4B beads (GE Healthcare, Chicago, IL, USA) were homogenously added to each well (of 96-deep well plates). The top opening was sealed tight using 96-well cap mats (Thermo Fisher Scientific, Waltham, MA, USA). The plates were sealed with cap mats (Thermo Fisher Scientific, Waltham, MA, USA) and incubated vertically on shaker with 80 rpm at 4 °C for 80 min for the binding of proteins to the GSH bead. These mixtures were carefully transferred to 96-well filter plates with 20 µm pore size (Thermo Fisher Scientific, Waltham, MA, USA) using wide-bore pipette tips. Wash buffer I and wash buffer II were added and centrifuged at 1000 rpm for 30 s simultaneously to remove impurities other than protein-GSH bead complex. The 50 µL elution buffer containing reduced GSH was added, and filter plates with bottoms sealed were shaken vigorously at 4 °C for 1 h. Finally, proteins were collected, placing the 96-well receiver plate below the filter plate, centrifugating at 4000 rpm for 2 min.

### 4.2. Fabrication of Saccharomyces Cerevisiae Proteome Microarrays

*Saccharomyces cerevisiae* proteome microarrays were fabricated following a previously reported protocol [27]. Briefly, using Liquidator 96 manual pipetting system (Mettler Toledo Rainin, LLC, Oakland, CA, USA), *Saccharomyces cerevisiae* purified proteins in 96-well plates were transferred to 384-well plates. High throughput CapitalBio SmartArrayer™ 136 microarrays contact printer (Capitalbio Corporation, Beijing, China) with 48 microarray pins were used for spotting (in duplicate) the entire ~5800 individual *Saccharomyces cerevisiae* proteins, and several landmark proteins on the aldehyde-coated glass slides to fabricate *Saccharomyces cerevisiae* proteome microarrays. Microarray pins can spot around 300 uniform protein spots by single sample uptake, and after each duplicate spotting of protein, the microarrays pins were washed using the optimized protocol to avoid contamination between the protein samples. Thus, in one batch, SmartArrayer^TM^136 microarray printer allows to fabricate, in total, one hundred *Saccharomyces cerevisiae* proteome microarrays. To ensure the quality of *Saccharomyces cerevisiae* proteome microarrays per batch, the fabrication procedures were performed in a cold room with controlled temperature (at 4 °C) and humidity (below 40%). After spotting of the entire *Saccharomyces cerevisiae* purified proteins and landmarks in duplicate, the fabricated *Saccharomyces cerevisiae* proteome microarrays were incubated at 4 °C, overnight, to facilitate the covalent interaction between the printed proteins (amine group) with the aldehyde group coated on the glass slides. These *Saccharomyces cerevisiae* proteome microarrays were placed in the slide box, vacuum-sealed, and stored at −80 °C. The shape, size, and uniformity for each protein spot on *Saccharomyces cerevisiae* proteome microarrays were confirmed by probing anti-GST/ anti-His antibody-DyLight^TM^ 550 (Rockland Immunochemicals Inc., Pottstown, PA, USA) on *Saccharomyces cerevisiae* proteome microarrays, followed by scanning of *Saccharomyces cerevisiae* proteome microarrays using LuxScan^TM^ (10K Microarray Scanner; CapitalBio Inc., Beijing, China).

### 4.3. Saccharomyces Cerevisiae Proteome Microarray Assays with Sub5

N-terminal biotin labeled (biotinylated) Sub5 was purchased from Kelowna International Scientific Inc. (Taipei, Taiwan). *Saccharomyces cerevisiae* proteome microarrays assays with biotinylated Sub5 were performed for systematical identification of protein targets of Sub5. Briefly, *Saccharomyces cerevisiae* proteome microarrays stored in −80 °C were immersed in 1× PBS-T (0.05% Tween 20) and incubated with shaking for 2 min at room temperature (RT) to remove the unbound proteins on *Saccharomyces cerevisiae* proteome microarrays. Then, *Saccharomyces cerevisiae* proteome microarray were blocked with 3% BSA in 1× PBS incubated with 50 rpm shaking speed for 1 h at RT. *Saccharomyces cerevisiae* proteome microarrays were washed with 1× PBS-T (incubated with 50 rpm shaking speed for 5 min at RT, one time. Biotinylated Sub-5 was probed on *Saccharomyces cerevisiae* proteome microarrays and incubation with 50 rpm shaking speed for 1 h at RT. To remove unbound Sub5, *Saccharomyces cerevisiae* proteome microarrays were washed with PBS-T and incubated with 50 rpm shaking speed for 5 min at RT, for one time. Furthermore, to detect the signal of bound biotinylated Sub5 as well as 6× His tag of *Saccharomyces cerevisiae* proteins, *Saccharomyces cerevisiae* proteome microarrays were probed with streptavidin-DyLight 650 and anti-His antibody-DyLight™ 550, respectively. *Saccharomyces cerevisiae* proteome microarrays were incubated with 50 rpm shaking speed for 1 h at RT. Later, *Saccharomyces cerevisiae* proteome microarrays were washed with PBS-T incubated with 50 rpm shaking speed for 5 min at RT, for three times. Finally, *Saccharomyces cerevisiae* proteome microarrays were dried (1000 rpm for 1 min) and scanned with LuxScan to detect the binding signals of Sub5 and 6× His on the *Saccharomyces cerevisiae* proteome microarrays.

### 4.4. Identification of Protein Targets of Sub5 from Saccharomyces Cerevisiae Proteome Microarrays

Using GenePix Pro 6.0 software, the scan files of Saccharomyces cerevisiae proteome microarrays probed with Sub5 were opened and the protein spots on *Saccharomyces cerevisiae* proteome microarray were align with their names. After alignment, the binding signals of biotinylated Sub5 and 6× His tag with immobilized *Saccharomyces cerevisiae* proteins on *Saccharomyces cerevisiae* proteome microarrays were exported as GPR files. In total, three GPR files obtained from triplicate *Saccharomyces cerevisiae* proteome microarrays assays were drag and opened with excel files. For the selection of potential protein targets of Sub5, the binding affinity data of Sub5 to *Saccharomyces cerevisiae* proteins from the triplicate *Saccharomyces cerevisiae* proteome microarrays were analyzed together by applying several statistical cutoff parameters. Firstly, the signal intensities of all of the protein spots on proteome microarrays assays were normalized using median scaling normalization. Secondly, the binding affinity of Sub5 to *Saccharomyces cerevisiae* proteins were selected to be greater than 100, and the coefficient variation (CV) between the duplicate protein spots on a single *Saccharomyces cerevisiae* proteome microarray to be lower than 0.5. Thirdly, to define the hits as positive, a further two cutoff criteria were applied. First, the binding intensity signal should be greater than local cutoff of two standard deviation (SD) above the signal mean for each spot. Second, the fold ratio of Sub5 signal to anti-His antibody signal should be greater than 0.5. The list of protein targets of Sub5 generated through the above-mentioned statistical cutoffs were individually checked with the eye from the significant signal in the triplicate *Saccharomyces cerevisiae* proteome microarrays. Finally, the proteins that passed the eye visualization test in all three proteome microarrays were selected as the protein targets of Sub5.

### 4.5. Bioinformatics Analysis of Gene Ontology

The Database for Annotation, Visualization, and Integrated Discovery (DAVID), version 6.8 (https://david.ncifcrf.gov/) [15], is an online platform with several bioinformatics tools. DAVID, being user friendly, was used to analyze the enrichment analysis for the proteins targets of Sub-5 obtained from *Saccharomyces cerevisiae* proteome microarrays. For the identification of enriched categories representing protein involved in similar functions and complexes, this investigation was necessary. These enrichments also provide the valuable insight to understand the mechanism of Sub5 from the identified proteins targets. DAVID online functional annotation tools include enrichment analysis in Gene Ontology (GO), showing enrichment in three major categories: biological process, cellular component, and molecular function. The protein families (PFAM) domain enrichment tool of DAVID was used to provide over-representation of the protein domain or motif analysis among the protein targets of Sub5. A stringent *p*-value cutoff ≤0.05 was applied to select only the significantly enriched terms in GO and PFAM categories.

### 4.6. Bioinformatics Analysis for Protein–Protein Interaction

To explore the protein–protein interaction of the protein targets of Sub5 obtained from *Saccharomyces cerevisiae* proteome microarrays, STRING (version 11.0) was used [16]. For the protein–protein interaction network, multiple protein search options were selected to include all of the 128 protein targets of Sub5. Furthermore, a stringent cutoff of high confidence 0.7 were deployed in the minimum required interaction score categories, to select only the significant interaction network.

### 4.7. Motif Search by MEME

To identify the consensus motif in the protein targets of Sub5, MEME Suite of motif-based sequence analysis tools (version 5.2.0) was used [17]. The Fasta files of all 128 protein targets of Sub5 were upload to the online analysis platform of MEME, using minimum and maximum motif width of 6 and 10, respectively, to obtain consensus motif in a maximum number of Sub5 protein targets.

## 5. Conclusions

In the current study, we identified, in total, 128 protein targets of Sub5 using *Saccharomyces cerevisiae* proteome microarrays. Bioinformatics analysis of the protein targets of Sub5 in GO biological process showed several significant enriched categories, the majority of these categories involved in actin-related processes. Moreover, eisosome filament, which provides membrane integrity and is involved in response to temperature stimulus, and works together with actin filament, showed enrichment in several categories under biological processes. These results indicated that Sub5 mostly targets to actin-related proteins and affects the cytoskeleton governing central role in morphogenetic alterations, along with cell division. Moreover, enrichment in “regulation of cell communication” and “regulation of signal transduction” showed diverse mechanism of Sub5 that were not related to actin. Consistent with GO biological process results, GO cellular component, and GO molecular function depicted major enrichment in actin-related components and functions, respectively. Similar results were observed in the PFAM domain enrichment analysis. STRING analysis also showed a separate interaction hub for actin-related proteins, whereas other interaction hubs were interconnected. Finally, MEME analysis identified a consensus motif of 10 amino acid sequences that are present in ~59% of protein targets of Sub5. Consistent with GO and STRING analysis, nine actin-related proteins were included in the MEME protein list. Thus, all of these findings indicate that Sub5 protein targets are highly enriched in actin-related proteins mechanism.

Taken together, the multiple protein targets of Sub5 identified in this study will be beneficial for looking into the complete mechanism of actions of Sub5, and develop a novel drug as an antifungal agent. Moreover, the targets of several potential AMPs that have high therapeutic values have not been identified. In the future, we will use this platform to identify the entire protein targets of other potential AMPs for understanding the mechanism of actions in terms of antibacterial or antifungal activities.

## Figures and Tables

**Table 1:** Enrichment categories in Gene Ontology (GO) biological processes for the protein targets of Sub5.

Term	Enrichment in Molecular Function	*p*-Value	Hit in This Category	Total Gene in This Category
GO:0030029	actin filament-based process	0.0000136	13	129
GO:0007015	actin filament organization	0.0000203	10	74
GO:0060255	regulation of macromolecule metabolic process	0.000494	40	1124
GO:0044396	actin cortical patch organization	0.000558	5	19
GO:0080090	regulation of primary metabolic process	0.000628	40	1137
GO:0031323	regulation of cellular metabolic process	0.000726	40	1145
GO:0048523	negative regulation of cellular process	0.001034	25	592
GO:0050790	regulation of catalytic activity	0.001206	19	392
GO:1902589	single-organism organelle organization	0.00121	23	528
GO:0007114	cell budding	0.00149	7	60
GO:0051301	cell division	0.00157	16	304
GO:0022607	cellular component assembly	0.00207	31	848
GO:0043254	regulation of protein complex assembly	0.00265	8	90
GO:0010646	regulation of cell communication	0.00844	9	139
GO:0033554	cellular response to stress	0.01015	25	705
GO:0043933	macromolecular complex subunit organization	0.01683	30	938
GO:0009408	response to heat	0.02003	6	75
GO:0009966	regulation of signal transduction	0.02036	8	132
GO:0022411	cellular component disassembly	0.02356	8	136
GO:0009266	response to temperature stimulus	0.02696	6	81
GO:0032231	regulation of actin filament bundle assembly	0.02925	3	13
GO:0007049	cell cycle	0.03250	25	782
GO:0007117	budding cell bud growth	0.03356	4	34
GO:0007163	establishment or maintenance of cell polarity	0.03434	7	116
GO:0009889	regulation of biosynthetic process	0.03619	29	956
GO:0006996	organelle organization	0.03937	46	1704
GO:0043170	macromolecule metabolic process	0.04550	77	3178
GO:0031324	negative regulation of cellular metabolic process	0.04558	16	447
GO:0051171	regulation of nitrogen compound metabolic process	0.04726	29	979
GO:0009892	negative regulation of metabolic process	0.04787	16	450

**Table 2:** Enrichment categories in GO cellular component for protein targets of Sub5.

Term	Enrichment in Molecular Function	*p*-Value	Hit in This Category	Total Gene in This Category
GO:0005938	cell cortex	0.000022	15	175
GO:0030427	site of polarized growth	0.000077	18	274
GO:0005937	mating projection	0.000131	12	133
GO:0042995	cell projection	0.00015	12	135
GO:0043332	mating projection tip	0.000219	11	118
GO:0043232	intracellular non-membrane-bounded organelle	0.000245	50	1429
GO:0044463	cell projection part	0.000425	11	128
GO:0044430	cytoskeletal part	0.000524	15	235
GO:0005933	cellular bud	0.008774	13	254
GO:0044428	nuclear part	0.00938	40	1260
GO:0005935	cellular bud neck	0.01021	11	197
GO:0044424	intracellular part	0.02049	127	5588
GO:0044427	chromosomal part	0.02443	17	433
GO:0005622	intracellular	0.02741	127	5603
GO:0070938	contractile ring	0.04085	3	15
GO:0005826	actomyosin contractile ring	0.04085	3	15
GO:0036286	eisosome filament	0.04297	2	2

**Table 3:** Enrichment categories in GO molecular functions for the protein targets of Sub5.

Term	Enrichment in Molecular Function	*p*-Value	Hit in This Category	Total Gene in This Category
GO:0019899	enzyme binding	0.000705	12	153
GO:0008092	cytoskeletal protein binding	0.0019716	9	100
GO:0032403	protein complex binding	0.0138161	7	86
GO:0042393	histone binding	0.0207646	5	46
GO:0019208	phosphatase regulator activity	0.049133	4	36

**Table 4:** Enrichment categories in protein domain analyzed by PFAM for the protein targets of Sub5.

	*p*-Value	Hit in This Category	Total Gene in This Category	Genes
Cytoskeletal-regulatory complex EF hand (EF hand represent helix E and F in parvalbumin)	0.001542	3	3	YBL047C, YNL084C, YIR006C
SRC homology 3 domain (SH3 domain)	0.011565	4	21	YER118C, YAR014C, YJL020C, YCR088W
Ubiquitin-conjugating enzyme	0.039661	3	14	YDR054C, YEL012W, YGL058W
Eisosome component PIL1 protein	0.045367	2	2	YGR086C, YPL004C
Tropomyosin like	0.045367	2	2	YNL079C, YIL138C

## Data Availability

Not applicable.

## Supplementary Materials

The following are available online at https://www.mdpi.com/1422-0067/22/2/760/s1, Figure S1: Enlarged protein image of 128 protein targets of Sub5 from the triplicate *Saccharomyces cerevisiae* proteome microarrays assay, Table S1: Two standard deviation (2SD) protein targets (i.e., 128 proteins) of Sub5 identified from *Saccharomyces cerevisiae* proteome microarrays, Table S2: 75 protein targets of Sub5 that have common motif identified from MEME analysis.
