# Supplementary material for: Systematic Identification of Protein Targets of Sub5 Using Saccharomyces cerevisiae Proteome Microarrays
Source: Int J Mol Sci. 2021 Jan 13;22(2):760. doi: 10.3390/ijms22020760 (PMC7828587; doi:10.3390/ijms22020760)
Supplement: Supplementary file 1 [file ijms-22-00760-s001.pdf]

Supplementary Table S1. Two standard deviation (2SD) protein targets (i.e. 128 proteins) of Sub-5 identified from *Saccharomyces cerevisiae* proteome microarrays. Protein with star mark (*) denote ATP or ATP-dependent proteins.

YOR239W	YJR092W	YGL153W	YLR055C	YLL048C*	YNL250W*	YGL207W	YDR103W
YCR088W	YBL011W	YBR296C	YEL012W*	YER096W	YKL052C	YKL181W*	YNL245C
YMR173W*	YMR311C	YLR114C	YJR072C*	YNR038W*	YJL107C	YER095W*	YNL084C
YBL047C	YNL186W	YDL153C	YDR054C*	YBR288C	YER118C	YJL051W	YEL056W
YJL115W	YKL028W*	YDR229W	YLR196W	YDL134C	YPL071C	YKL159C	YKL074C
YKL195W	YCL054W	YER116C	YGL058W*	YJR049C*	YKR048C	YAL003W	YOR354C
YIL138C	YMR039C	YJR125C	YOL054W	YJL123C	YDR264C	YIL154C	YDR152W
YLL008W*	YGR086C	YMR091C*	YDR273W	YGL161C	YOL039W	YLR340W	YGL256W
YDR068W	YHR158C	YGR202C	YGR179C	YGL130W	YOR340C	YNL175C	YMR291W*
YML062C*	YLL026W	YPL217C*	YCL029C	YDR468C	YDR346C	YDR188W*	YBR092C
YCL043C	YLR295C*	YPL004C	YLR435W	YMR235C	YPL023C	YHR205W*	YER107C
YJL020C	YDL223C	YIR006C	YLR257W	YGL134W	YER030W	YGR013W	YMR074C
YNL079C	YFL034W	YKL117W	YDR168W	YDR496C	YER148W	YNL099C	YFR009W*
YPL190C	YHR171W	YJL098W	YMR260C	YPR152C	YCR052W*	YKL073W*	YKL160W
YKR090W	YGL208W	YDR382W	YHR156C	YGL073W	YEL051W*	YAR014C	YKL040C
YFR015C	YGL090W	YIR003W	YKL081W	YGR187C	YOR194C	YJL065C*	YBL046W

Supplementary figure S1.

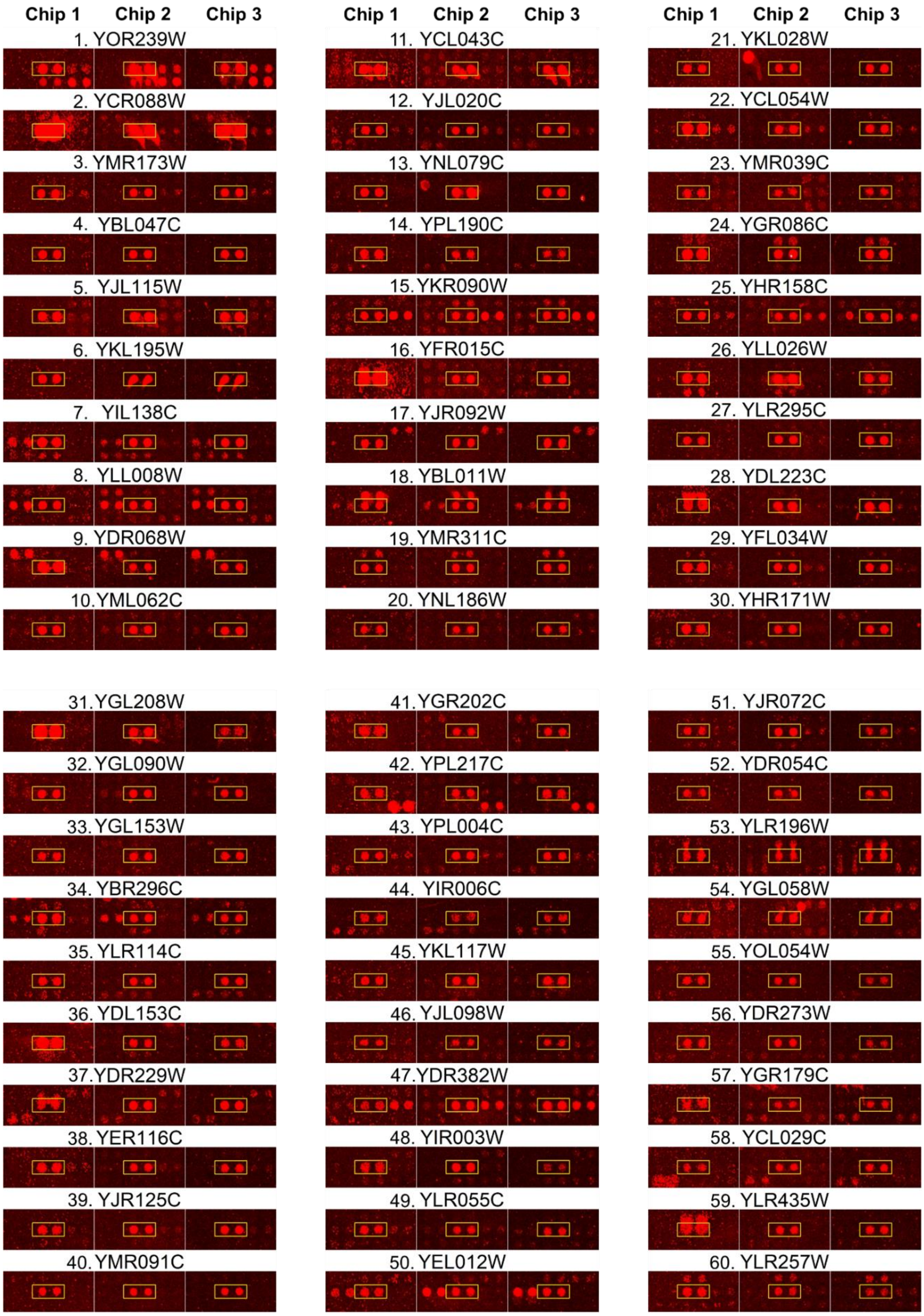

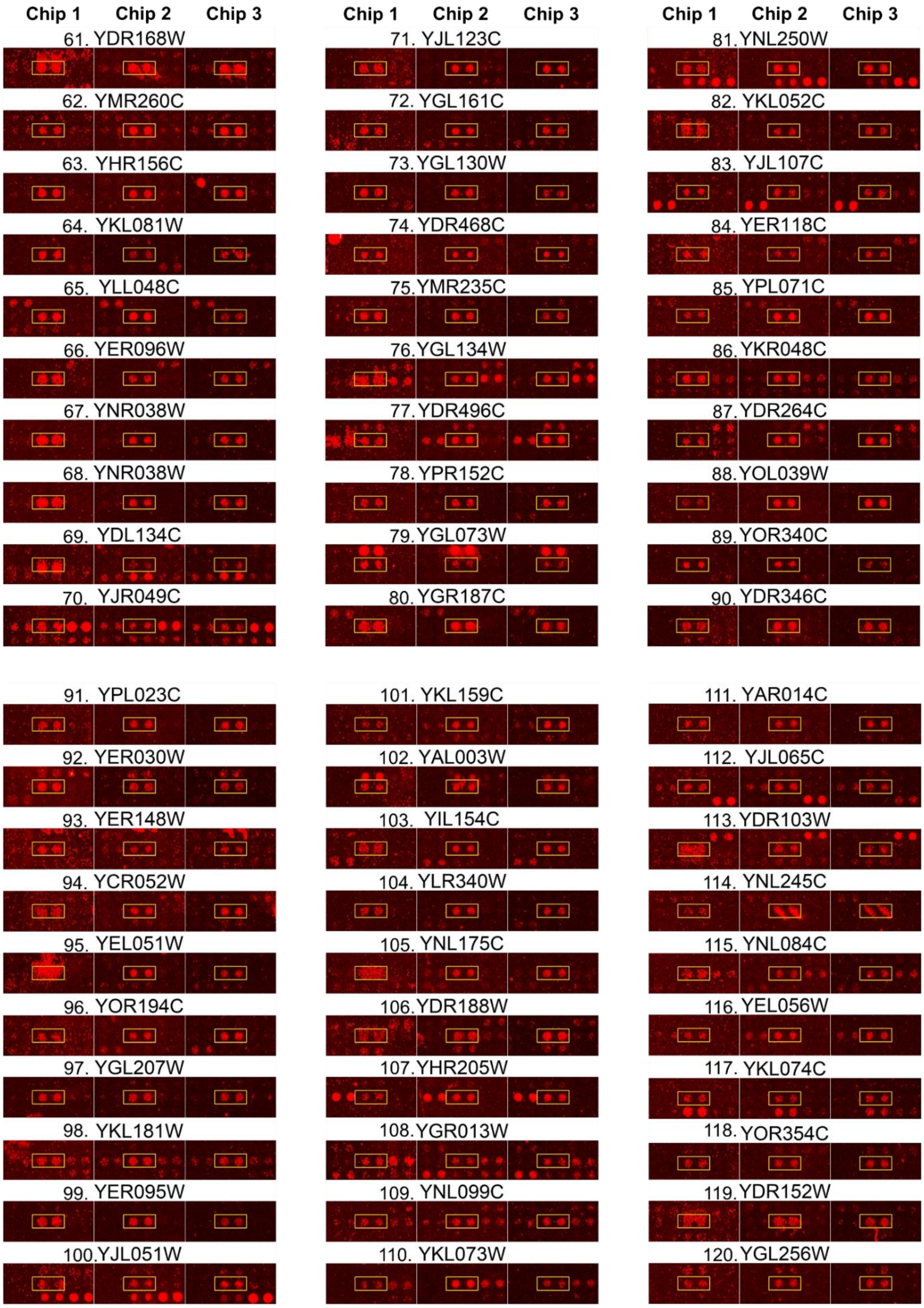
Chip 1 Chip 2 Chip 3
61. YDR168W
62. YMR260C
63. YHR156C
64. YKL081W
65. YLL048C
66. YER096W
67. YNR038W
68. YNR038W
69. YDL134C
70. YJR049C
71. YJL123C
72. YGL161C
73. YGL130W
74. YDR468C
75. YMR235C
76. YGL134W
77. YDR496C
78. YPR152C
79. YGL073W
80. YGR187C
81. YNL250W
82. YKL052C
83. YJL107C
84. YER118C
85. YPL071C
86. YKR048C
87. YDR264C
88. YOL039W
89. YOR340C
90. YDR346C
91. YPL023C
92. YER030W
93. YER148W
94. YCR052W
95. YEL051W
96. YOR194C
97. YGL207W
98. YKL181W
99. YER095W
100. YJL051W
101. YKL159C
102. YAL003W
103. YIL154C
104. YLR340W
105. YNL175C
106. YDR188W
107. YHR205W
108. YGR013W
109. YNL099C
110. YKL073W
111. YAR014C
112. YJL065C
113. YDR103W
114. YNL245C
115. YNL084C
116. YEL056W
117. YKL074C
118. YOR354C
119. YDR152W
120. YGL256W

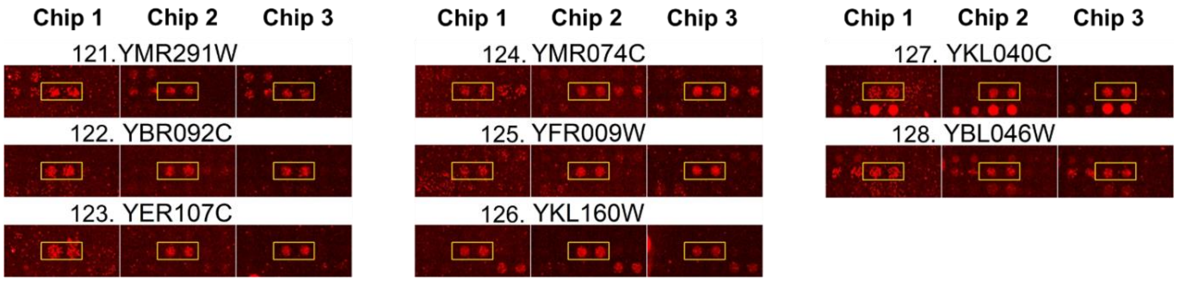

Supplementary figure S1. Enlarged protein image of 128 protein targets of Sub-5 from the triplicate *Saccharomyces cerevisiae* proteome microarrays assay. Individually enlarge image of 128 protein targets of Sub-5 identified from the triplicate *Saccharomyces cerevisiae* proteome microarrays assays. Each protein is spotted in duplicate on *Saccharomyces cerevisiae* proteome microarrays and *Saccharomyces cerevisiae* proteome microarrays assay of Sub-5 probing were performed in triplicate. Protein targets identification of Sub-5 were statistically analyses using several cutoff parameters and finally each protein was analyzed by eye-bowling to confirm obvious signal of these proteins on triplicate *Saccharomyces cerevisiae* proteome microarrays. Red spots represent signal of Sub-5 bound to immobilized Saccharomyces cerevisiae proteome

Supplementary Table S2. 75 protein targets of Sub-5 that have common motif identified from MEME analysis

YJL123C	YDR068W	YIR006C	YOR194C	YDR054C
YPL190C	YKR048C	YML062C	YGL207W	YNR038W
YKL028W	YPL004C	YDR382W	YOR239W	YJL065C
YBL011W	YGR013W	YDR273W	YEL056W	YEL012W
YDL153C	YKL117W	YJL020C	YBL046W	YLR295C
YOL054W	YNL099C	YLR340W	YLR196W	YMR039C
YPR152C	YDR152W	YIL154C	YLR114C	YCR052W
YJL115W	YMR260C	YGL058W	YAL003W	YIL138C
YLL008W	YER030W	YDR103W	YMR091C	YGL090W
YGR187C	YJR125C	YJL098W	YGR202C	YMR311C
YMR235C	YOL039W	YGL073W	YCL043C	YER095W
YGR086C	YLR055C	YDR496C	YKL181W	YNL079C
YNL186W	YJR092W	YGR179C	YDR468C	YGL134W
YDR346C	YLR257W	YKL160W	YPL023C	YMR074C
YCR088W	YLR435W	YPL217C	YAR014C	YER116C
